# Updated Insight into the Physiological and Pathological Roles of the Retromer Complex

**DOI:** 10.3390/ijms18081601

**Published:** 2017-07-25

**Authors:** Yakubu Saddeeq Abubakar, Wenhui Zheng, Stefan Olsson, Jie Zhou

**Affiliations:** 1 State Key Laboratory of Ecological Pest Control for Fujian and Taiwan Crops, College of Life Science, Fujian Agriculture and Forestry University, Fuzhou 350002, China; ay.saddeeq@gmail.com (Y.S.A.); stefan@olssonstefan.com (S.O.); 2 College of Plant Protection, Fujian Agriculture and Forestry University, Fuzhou 350002, China

**Keywords:** retromer, receptors, vacuolar protein sorting, endosome, disease

## Abstract

Retromer complexes mediate protein trafficking from the endosomes to the *trans*-Golgi network (TGN) or through direct recycling to the plasma membrane. In yeast, they consist of a conserved trimer of the cargo selective complex (CSC), Vps26–Vps35–Vps29 and a dimer of sorting nexins (SNXs), Vps5–Vps17. In mammals, the CSC interacts with different kinds of SNX proteins in addition to the mammalian homologues of Vps5 and Vps17, which further diversifies retromer functions. The retromer complex plays important roles in many cellular processes including restriction of invading pathogens. In this review, we summarize some recent developments in our understanding of the physiological and pathological functions of the retromer complex.

## 1. Introduction

Exchange of materials between a cell and its surrounding is essential for cellular functions. These interactions are strictly regulated, depending on the physiological and/or pathological conditions of the cell. Extracellular macromolecules are taken up into the cell by a process called endocytosis, whereby a portion of the plasma membrane traps the molecules and invaginates into the cytoplasm to form an endocytic vesicle. At this stage, some plasma membrane-localized proteins appear as membrane components of the transport vesicles. These internalized vesicles are directed to the cellular sorting station, the early endosome, where they fuse with the endosomal membrane to deliver their contents. Cargoes that need to be recycled (such as the plasma membrane proteins) are sorted out from the endosomal compartment for transport to their appropriate destinations whereas those destined for degradation are retained in the endosomal lumen and delivered to the lysosome/vacuole. A similar but oppositely directed process termed exocytosis occurs to balance the cellular composition and relocalization of some cell components. These events have been addressed in some excellent reviews [1,2]. Protein sorting machineries such as clathrin, SNX4/41/42, and the retromer complex are usually involved in the endosomal sorting of cargo proteins [2,3,4,5,6,7,8]. These machineries, together with their regulators, form distinct pathways that ensure proper delivery of proteins to their appropriate destinations.

The retromer complex was first discovered in yeast as a heteropentameric protein complex involved in the retrieval of carboxypeptidase Y (CPY) receptor, a vacuolar protein sorting (Vps10) protein, from endosome to trans-Golgi network (TGN) [9]. Vps10 is localized to the TGN, where it binds newly synthesized CPY and delivers it to the endosome for subsequent transport to the vacuole where it functions as an acid hydrolase. Cargo-less Vps10 needs to be transported back to the TGN after the delivery of CPY for additional rounds of the transport, and the retromer complex plays a role here by sorting Vps10 from the endosome for retrograde transport to the TGN; otherwise it would be transported to the vacuole and be degraded [4,10]. From its discovery to date, retromer complex has been explored structurally, functionally and, to some extent, mechanistically. In addition to the Vps10 sorting, the complex has also been shown to mediate the trafficking of many other cargoes, channeling them to pathways other than the endosome-to-TGN trafficking route [11,12,13,14,15,16,17,18,19]. In yeast, retromer is composed of five distinct proteins including the Vps26-Vps35-Vps29 trimer and a sorting nexin (SNX) dimer consisting of Vps5 and Vps17 [9]. The trimer is well known for its cargo sorting property on the endosomal membrane and, as such, it is often referred to as cargo selective complex (CSC). This retromer subcomplex is highly conserved among eukaryotes, making it the core retromer subcomplex with Vps35 interacting with Vps26 and Vps29 at its N- and C-termini respectively [9,20,21]. Sometimes, this trimer may be involved in cargo sorting independent of the SNX dimer such as in *Arabidopsis* [22]. On the other hand, Vps5 and Vps17 are SNX proteins containing Bin-Amphysin-Rvs (BAR) domains for inducing membrane curvature during cargo sorting, and phox homology (PX) domains that anchor them to the endosomal membranes via strong interaction between the PX domains and the phosphatidylinositide-3-phosphates (PI(3)P) of the membrane [1,21]. However, the architectural and functional properties of the retromer seem to be more complicated in higher eukaryotes. For instance, mammals possess two orthologs each of Vps5 and Vps17: SNX1/SNX2 and SNX5/SNX6, respectively. Additionally, mammalian CSC can interact with some other SNX proteins, apart from the aforementioned ones, to form a fully functional retromer complex that recognizes different cargoes. In this review, we summarize the current understanding of the structure and function of the retromer complex and its roles in disease and development that make it a central protein sorting machinery in eukaryotes.

## 2. Current Overview of the Knowledge of the Composition of Retromer Complex

### 2.1. The Cargo Selective Complex (CSC)

Although the retromer complex appears to be conserved in eukaryotes, certain differences in its subunits exist especially in higher eukaryotes, leading to differences in cargo recognition and binding sites. The subunits of the conserved CSC may have different homologues, respectively, in the same or different species. For instance, yeasts do not have paralogs of Vps26, Vps29, and Vps35; mammals, however, have two distinct but highly similar isoforms of Vps26: Vps26A and Vps26B [23,24,25]; *Arabidopsis thaliana* also possesses these two *VPS26* genes (A and B) and three other genes encoding Vps35A, Vps35B, and Vps35C, respectively [26]. One homologue may be functionally distinct from the other as mutations in mammalian Vps26A, but not Vps26B, are linked to atypical Parkinsonism [27,28]. Furthermore, Vps26A-retromer has been implicated in mediating the trafficking of mitochondria-derived vesicles (MDVs) that convey mitochondrial-anchored protein ligase (MAPL) from mitochondria to peroxisomes in mammals [29]. Again, retromer-mediated trafficking of cation-independent mannose-6-phosphate receptor (CI-MPR) is only dependent on Vps26A-retromer. This functional difference is due largely to the presence of a C-terminal tail on Vps26B, the deletion of which enables Vps26B to interact with exogenous CI-MPR [30]. Protease-activated receptor 2 (PAR-2) is a plasma membrane-localized member of G-protein-coupled receptor (GPCR) family that regulates the level of intracellular calcium in a trypsin-dependent pathway, after which it is channeled to the lysosome for degradation [31,32]. Repopulation of the membrane PAR-2 level for continued cell signaling may be achieved via de novo synthesis [33]. A recent study established a novel role for the Vps26B-retromer in regulating the trafficking of PAR-2 to plasma membrane for repopulation in human embryonic kidney (HEK293) cell lines [34]. Whether or not the C-terminal region of Vps26B is the key determinant of PAR-2 recognition as a cargo awaits future investigation. In *Arabidopsis*, however, the two VPS26 isoforms seem to be redundant as *vps26A* and *vps26B* deletion mutants were phenotypically similar to the wild type, respectively [35]. So far, isoforms of *VPS35* (*VPS35A*, *VPS35B*, and *VPS35C*) have only been reported in plants (particularly in *Arabidopsis*). Single mutants of any of these three isoforms, as well as *vps35Avps35C* double mutants, were reported by Yamazaki et al. [36] to have no obvious defects as compared to the wild type. The authors, however, generated a *vps35SBvps35C* double mutant that was phenotypically dwarf and showed early leaf senescence, signifying that *VPS35B* is responsible for leaf senescence retardation and that *VPS35A* and *VPS35C* together can take care of the functions of *VPS35B* in the *vps35B* mutant. To date, *VPS29* has never been reported to have any homologue. In addition to cargo sorting, the CSC is used as a platform through which some endosome regulating proteins (such as strumpellin) are recruited to the endosomal membrane for their functions [37]. Retromer functions may therefore be altered by different homologs of the CSC subunits.

### 2.2. Sorting Nexins Define Distinct Retromer Complexes

Sorting nexins, on the other hand, provide even more retromer diversity in terms of cargo recognition and trafficking pathway. In this regard, three distinct forms of retromer complex are recognized: SNX-BAR-retromer, SNX3-retromer, and SNX27-retromer.

**Figure 1:**
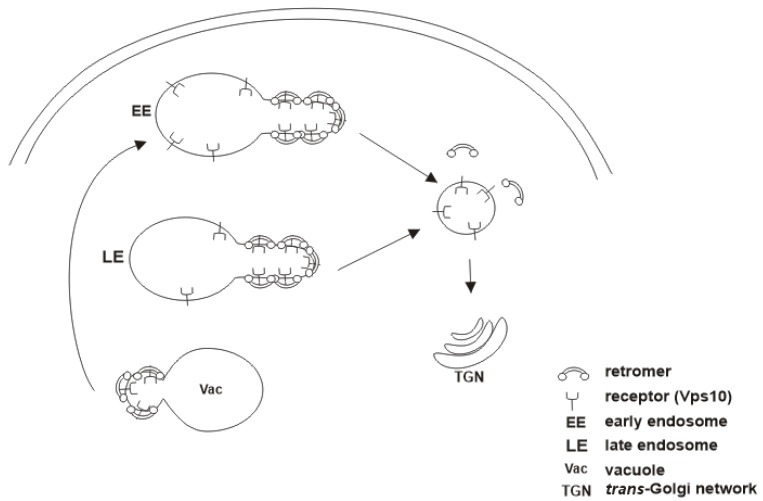
Schematic representation of retromer-mediated Vps10 sorting at the vacuole. Missorted Vps10 cargoes at the endosomes reach the vacuole and are rescued from degradation via retromer-mediated sorting at the vacuolar membrane and delivered to the early endosome for further sorting to the Golgi.

SNX proteins containing BAR domains could be termed SNX-BAR proteins, some of which interact with the CSC to form the SNX-BAR-retromer complex [38]. Vps5 and Vps17 in yeast and the mammalian SNX1/2 and SNX5/6 are SNX-BAR subunits of the retromer [8,9]. Their BAR domains are required for induction of membrane curvature to generate transport vesicles for trafficking of retromer cargoes [39,40]. In addition to the BAR domains, these SNX proteins also possess PX domains that enable them target and bind PI(3)P on endosomal membranes [19,41]. Although mammalian CI-MPR binds directly to Vps35, this binding is absolutely dependent on a conserved groove on SNX5, making the SNX-BAR-retromer essential for prevention of lysosomal degradation of CI-MPR [42,43]. Furthermore, SNX5/6 subunits of the retromer interact with the largest dynactin subunit, p^150glued^, which recruits the microtubule-dependent dynein/dynactin motor complex to retromer-associated vesicles for transport [8,43]. Interestingly, a recent report indicated that retromer also sorts “leaked” Vps10 cargo at the yeast vacuole and transports it back to the endosome for normal trafficking to the Golgi [44]; (Figure 1). The results indicated that this vacuolar sorting seems to be dependent on the SNX-BAR-retromer complex. In *Arabidopsis*, a microtubule-associated protein, CLASP (cytoplasmic linker associated protein), requires an SNX-BAR-retromer to promote the recycling of PIN2, an auxin efflux protein [45]. In *Caenorhabditis elegans*, the SNX-BAR-retromer prevents the lysosomal degradation of the cell surface receptor CED-1, which is essential for apoptotic cell clearance [46,47]. Sometimes, however, some retromer-associated SNX-BAR proteins may function independently to mediate important physiologic functions such as the lysosomal degradation of activated PAR1 (protease-activated receptor-1) [48,49] and EGFR (epidermal growth factor receptor) [50], and regulation of purinoceptor 1 (P_2_Y_1_) recycling to the cell surface [51].

SNX3 is a PX-only sorting nexin (reviewed in [1,52]) that interacts with the CSC via direct binding to Vps35, forming an SNX3-retromer that functions independent of SNX-BARs [53,54,55]. This form of retromer has been shown to mediate the trafficking of Wntless (Wls), a receptor that binds and transports the Wnt protein, from the early endosome to the TGN [54,56]. Mutating Vps29 in the SNX3-retromer partially disrupts the retrieval of Wls, resulting in their accumulation at the late endosome [57]. Interestingly, this report further established that the accumulated Wls at the late endosomes of the *vps29* mutants can be retrieved by SNX-BAR-retromer, indicating an overlap in cargo recognition of the two retromer forms. Retromer-associated SNX-BARs are localized to both the early and late endosomes. Whether SNX-BAR-retromer sorts Wls in the presence of a fully functional SNX3-retromer remains to be investigated. Another research group working with *Drosophila* reported the involvement of SNX3-retromer and SNX6-(an SNX-BAR) retromer in the sorting and trafficking of lysosomal enzyme receptors back to the Golgi, after delivering the enzymes [58]. These observations pose a question on how these two distinct forms of retromer achieve cargo specificity. Further investigations of the molecular mechanisms of operation of the two retromers will shine more light on this question. Recently, (F/Y)E(F/L) has been reported as a consensus sorting sequence for SNX3-retromer cargoes [59].

Grd19 is a yeast homologue of SNX3 that also interacts with the CSC to form a functional Grd19-retromer complex that regulates the endosome-to-Golgi trafficking of the Fet3-Ftr1 heterodimer, a plasma membrane-localized reductive iron transporter [60]. In addition to this cargo, Grd19-retromer also mediates the trafficking of other cargoes including Kex2, Ste13, and Pep12 [3,61]. Like SNX-BARs, SNX3 is also involved in retromer-independent cargo sorting [62].

**Figure 2:**
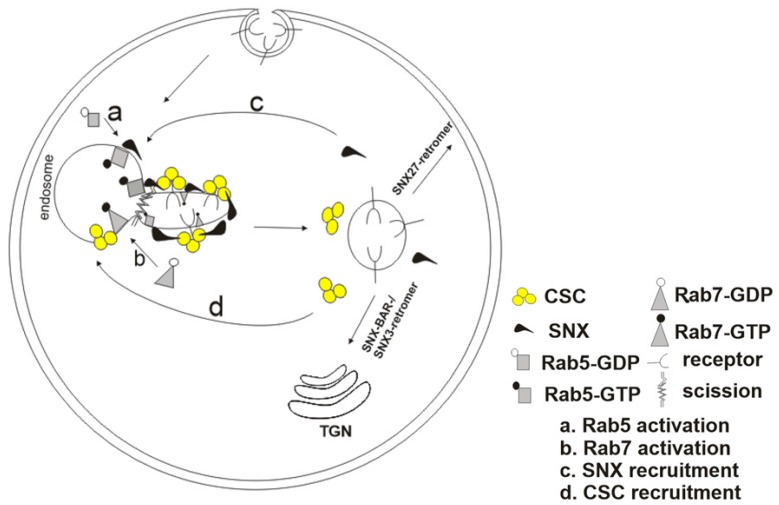
Recruitment of retromer to the endosome and the fission of the vesicle after its exit from the endosome. Activated Rab5 and Rab7 (Rab5-GTP and Rab7-GTP, respectively) recruit the SNX and CSC subcomplexes, respectively, to the endosomal membrane. The retromer subcomplexes assemble into a complete retromer complex and concentrate cargoes following membrane tubulation by the SNX proteins, and subsequent vesicle scission. Further fission of the vesicle occurs downstream of its endosome exit for cargoes taking different itineraries.

While SNX-BAR- and SNX3-retromers channel their cargoes into the endosome-to-Golgi trafficking pathway, SNX27-retromer mediates direct endosome-to-plasma membrane trafficking without passing through the TGN [63,64,65]. SNX27 is a FERM (four-point-one, ezrin, radixin, moesin) domain-containing sorting nexin that recognizes and binds NPxY motifs on its cargoes [66]. Unlike the SNX-BAR proteins, SNX27 lacks the BAR domain for induction of membrane curvature and this makes it still unclear how membrane curvature is achieved by sorting nexins. In addition to the FERM domain, SNX27 also has a PDZ (post-synaptic density-95/disc large/zonaoccludens) domain that recognizes C-terminal PDZ ligands on some cargoes as sorting signals for direct endosome-to-plasma membrane recycling [67,68]. SNX27 was found to associate with Vps26, although immunoprecipitation and SILAC (stable isotope labeling using amino acids in cell) proteomics also revealed that this sorting nexin interacts with SNX-BAR-retromer [69]. A model proposed in this report suggested that some cargoes sorted by the SNX27 could be delivered to SNX-BAR positive vesicles for SNX-BAR-retromer-mediated transport to their destinations. However, suppression of retromer-associated SNX-BARs had an insignificant effect on the surface localization of these cargoes, suggesting that other carriers might also be involved in this transport. Varandas et al. [7] discovered that retromer cargoes destined for different itineraries (recycling/retrograde pathways) leave the endosomal membrane in a shared vesicle, after which further sorting of the cargoes and fission of the vesicle into their respective itineraries occur downstream of the endosome exit (Figure 2). This may provide a clue to why non-BAR domain containing sorting nexins such as the SNX3 and SNX27 may not need to be able to create membrane curvature for cargo orchestration.

How SNX27-retromer cargoes are directed to the plasma membrane and prevented from being trafficked to the Golgi still remains a difficult question. A recent work investigated this directionality and reported its dependence on interactions of the SNX27-retromer with FAM21 (family with sequence similarity-21) tail of the WASH (Wiskott–Aldrich syndrome protein and SCAR homology) complex [67]. The study elucidated that FAM21 controls the level of PI(4)P, which results in the dissociation of cargoes at the Golgi apparatus. A previous study [43] indicated that FAM21 interacts with Vps35 and that Vps35 (D620N) mutation partially interferes with this interaction, which reduces the efficiency of the endosome-to-TGN trafficking of CI-MPR (an SNX-BAR-retromer cargo) but not the endosome-to-plasma membrane recycling of the glucose transporter GLUT-1 (an SNX27-retromer cargo). Furthermore, depletion of the WASH complex components, strumpellin and WASH1, had no obvious consequences on SNX27-retromer routing, in contrast to depletion of FAM21 [64]. On the other hand, CI-MPR routing may depend on these subunits whose conformations may be affected by the Vps35 (D620N) mutation. Again, Vps35 (R524W) impairs the interaction of the retromer with its regulating machineries, which delays retrograde trafficking but had no obvious trafficking effect on SNX27-retromer-dependent recycling [70]. Recently, a novel protein, ANKRD50 (ankyrin repeat domain containing protein), was shown to be an essential part of the SNX27-retromer-WASH supercomplex [67]. The study indicated that the protein interacts with the N-terminus of FAM21, C-terminus-localized PDZ binding domain of SNX27 and Vps29 (around its E1300 amino acid residue). It was further shown that binding of ANKRD50 to the PDZ domain of SNX27 blocks the interaction of the SNX27-retromer with its cargoes (such as GLUT-1) that depend on the PDZ domain for their recycling, suggesting that the protein might be recruited by the SNX27-retromer-WASH only when the cargoes are needed to be released into recycling vesicles for transport to the plasma membrane.

Parathyroid hormone receptor (PTHR) is a class-B GPCR of bone cells and plays an important role in bone remodeling [71]. Stimulation of this receptor by parathyroid hormone (PTH) agonist induces PTHR internalization (by clathrin in association with β-arrestins) to the early endosome, where the agonist dissociates from the receptor in the acidic environment and the receptor is recycled [72,73,74,75,76]. Two independent research groups established that PTHR contains a PDZ-binding ligand that makes it a suitable SNX27-retromer cargo for recycling [63,77]. Mutation of the PDZ ligand of PTHR disrupted its interaction with the SNX27-retromer but did not affect the relocalization of the receptor to the plasma membrane signifying that the PTHR may also interact directly with the CSC in addition to its SNX27 binding [77], or be recycled via an additional pathway. Independent of its PDZ ligand, PTHR was confirmed from the latter report to interact with the CSC via direct binding to Vps26. The second hypothesis also seems positive from other investigations, which confirmed the retromer-dependent endosome-to-TGN trafficking of this receptor, which, with the current understanding of retromer pathways, is not associated with SNX27-retromer routing [14,78]. Similarly, β_1_-adrenergic receptor (β_1_-AR) recycling is SNX27-retromer-dependent through PDZ domain binding [79], but can also be sorted via an alternative sorting signal, protein kinase A (PKA)-substrate phospho-Ser^312^, independent of SNX27 [68,80]. Therefore, SNX27-retromer might have evolved to take care of fast recycling of some retromer cargoes.

Generally, there is growing evidence to establish the involvement of both the CSC and SNX subunits in cargo binding, contrary to the classical perception of relating cargo binding to only CSC, which resulted in it being called the “cargo selective/recognition complex”. In fact, CSC association with different kinds of SNX proteins contributes largely to the diversity of retromer cargoes, with the SNX proteins acting more or less as adaptor molecules.

## 3. Recruitment of the Retromer Complex Components to Endosomal Membranes

Retromer is not recruited to the endosomes as a complete retromer complex; rather, the CSC and the SNX subcomplexes are recruited separately, after which they assemble into a complex on the endosomal membranes [81,82,83,84]. Moreover, the CSC components may either assemble in the cytoplasm into a trimer before its recruitment or be recruited individually and get assembled on the membranes [20]. The small guanosinetriphosphatases (GTPases), Rab5 and Rab7, play crucial roles in endosomal recruitment of the SNX and CSC subcomplexes, respectively (Figure 2). However, the recruitment of CSC is partially dependent on Rab5 since overexpression of Rab5 causes dissociation of Vps26 from the endosome [85]. The already-recruited SNX subcomplexes also contribute to the recruitment of the CSC via interaction with cytoplasmic Vps35 and Vps29 [86,87]. After the recruitment of all the subunits, Rab5 dissociates and is replaced by more Rab7 along the early-to-late-endosome maturation pathway [88]. To retain the SNX subcomplexes on the endosome after Rab5 dissociation, PI(3)K (phosphatidylinositol-3 kinase)-dependent phosphorylation produces more PI(3)P, to which the SNX proteins bind [85]. However, Rab5 homologues in *Arabidopsis* are dispensable for CSC recruitment to the endosomal membrane [89].

Rab7 has higher affinity for Vps35 than for Vps26, and the attraction becomes stronger when Vps35 is already in interaction with Vps26 prior to its recruitment [82]. A conserved motif, PRLYL (or PRMYL in *Arabidopsis*), on Vps35 has been shown to be essential for its N-terminal interaction with Vps26 [20]. The components of the retromer complex get completely assembled on the endosomal membrane to initiate tubulation and cargo sorting. Rab7 function is also required for the fusion of late endosomes with lysosomes [90]. A research group identified the protein TBC1D5 (a GTPase activating protein), which interrupts CSC–Rab7 interaction to trigger the release of CSC from Rab7, enabling Rab7 to regulate the endosome–lysosome fusion [91]. This may be achieved by stoichiometric binding of TBC1D5 to CSC to form a TBC1D5–CSC, complex, which seems to be the most stable of all CSC interacting intermediates, thereby mediating the timely membrane coating and uncoating of the retromer complex essential for cargo trafficking [92]. However, more work is required to address the mechanistic relationships of the TBC1D5 and other proteins that regulate retromer functions and to uncover the factor(s) mediating the dissociation of the TBC1D5–CSC complex during/after retromer uncoating needed to pave the way for another round of retromer sorting.

## 4. Regulation of Retromer Recruitment and Vesicle Scission

Rab GTPases, particularly Rab5 and Rab7, are essential for retromer recruitment and assembly on the endosomes as well as for membrane tubulation [85,91]. An investigation in yeast demonstrated that Ypt7 (a yeast homologue of Rab7) plays a central role in tubule formation of SNX-BAR-retromer [93]. The GTPase is activated by the guanine-nucleotide exchange factor (GEF) Mon1-Ccz1 for CSC recruitment and assembly with SNX-BAR. This leads to membrane tubulation and subsequent movement of cargoes into the tubule and finally dissociation of the CSC from Ypt7. According to the authors, this makes Ypt7 available for interaction with HOPS tethering complex to initiate endosome-vacuole SNARE-dependent fusion. Fully formed retromer-coated tubules are then detached from the endosomal membranes for transport to appropriate destinations. However, the mechanism of the vesicle scission is not adequately understood. A publication by Gomez and Billadeau [24] suggested a model in which WASH complex mediates tubule scission through generation of F-actin force essential for the scission process. WASH promotes the budding of endosomal tubules by exerting pressure on the endosomal membranes in an opposite direction to a corresponding pulling force generated by microtubule-dependent molecular motors (such as dynamin) on the vesicle, which leads to vesicle scission [94,95]. A number of studies have shown the recruitment of WASH by retromer complex through direct interaction of Vps35 with FAM21 via a set of 21 leucine-phenylalanine-acidic (LFa) motifs on FAM21 [96,97,98,99]. Seaman’s research group also provided evidence supporting the WASH-mediated vesicle scission and further indicated that Vps35–Vps29 (but not Vps35–Vps26) interaction is essential for retromer recruitment of WASH complex [100]. The retromer–WASH interaction is also essential for endosomal recruitment of some retromer regulatory proteins such as ANKRD50, SDCCAG3, and RME-8 [43].

In another development, Burd’s group generated data elucidating that Vps1 and Mvp1 (a yeast orthologue of human SNX8) are required for the fission of SNX-BAR decorated endosomal vesicles where Vps1 promotes the fission events whereas Mvp1 is necessary for association of Vps1 with the endosome [39,101]. Spastin, a microtubule-severing protein, has also been found to promote the budding of endosomal tubules by generating microtubule plus ends, which in turn lead to an increase in the pulling force required for the budding [102]. This report also found that spastin function requires interaction with IST1, a component of the endosomal sorting complex required for transport (ESCRT). The endosomal recruitment of the retromer complex and vesicle scission appear to be complex, not yet fully understood processes requiring the combined functions of network of different proteins.

## 5. Regulation of the Retromer Complex Activity

The retromer complex is a physiologically important sorting machinery that controls the membrane protein composition of some intracellular organelles and the plasma membrane. For this, the activities of this protein complex should be tightly regulated along one pathway or the other. Further studies are needed to fill the gaps in our knowledge of the mechanisms of regulation of retromer functions. This could be important for the successful tackling of retromer dysfunction-related diseases (discussed below). Quite a few proteins, however, have been reported to regulate retromer functions in one way or the other. Endosome-to-Golgi retromer function, for example, is stabilized by Eps15 homology domain-containing protein-1 (EHD1), the RNAi suppression of which inhibits retromer function in this pathway [103]. An ADP ribosylation factor-6 (Arf6) has recently been implicated in regulating retromer tubule dynamics in mouse embryonic fibroblasts [104]. The report indicated that depletion of Arf6 results in aberrant retromer tubulation leading to mistrafficking of CI-MPR. As earlier stated, TBC1D5 regulates CSC–Rab7 interaction by triggering the release of CSC from Rab7 at the late endosome, thus controlling the CSC recruitment and release for normal retromer function [91]. Additionally, retromer cargo specificity is regulated in part by two WD40 domain-containing proteins, Ere1 and Ere2, which together interact with the retromer to enable it to recognize Can1 (a plasma membrane arginine transporter) as a cargo for transport from endosome back to the plasma membrane via the TGN [5]. The release of SNX27-retromer cargoes into recycling vesicles has earlier been said to be regulated by ANKRD50 [67]. Moreover, chitin synthase 3 (Chs3) recycling is retromer-dependent through binding of Vps26 to a YYL sorting signal of the enzyme [105]. This binding has recently been shown to be regulated by phosphorylation of the 6 loop of Vps26 via Mih1 signaling (Mih1 is a yeast homologue of Cdc25) [106]. Mih1 signaling is in turn regulated by protein kinase C, the ultimate mediator of TGN-plasma membrane transport of Chs3 [107,108]. In essence, regulation of retromer function occurs at different levels and depends on different interacting factors.

## 6. Roles of the Retromer in Cell Signaling and Autophagy

The fact that the retromer complex sorts and mediates the transport of diverse cargoes makes it essential for normal operation of many pathways. Its involvement in recycling signaling receptors such as β_2_-ARs [65] after ligand-induced endocytosis has received great attention from researchers interested in dissecting its roles in cell signaling. However, this area of retromer research is still in its infancy, though tremendous progress has been gained recently. Retromer influences PTH-PTHR generated cAMP signaling by recycling PTHR from the early endosome after its dissociation from β-arrestin, resulting in a switching off of this signaling pathway [14,78]. This role has recently been shown to be achieved through binding of the PDZ domain of SNX27-retromer to its ligand on the receptor for recycling [63]. The SNX27-retromer also recycles the interferon receptor 2 (IFNAR2) subunit of the IFNAR after its internalization. By doing so, the retromer is able to regulate not only the termination of JAK/STAT signaling induced by type I interferons but also its gene transcription [11]. Similarly, the Vps35/retromer turns off RANK (receptor activator of NF-κB) signaling in osteoclast lineage cells by trafficking RANK to Golgi necessary for normal bone deposition and resorption [109]. Another study reported the involvement of the retromer in nucleotide binding-leucine-rich repeat (NB-LRR)-mediated hypersensitive response signaling, which triggers autophagy in *Arabidopsis* [110], though it is still unclear how the retromer functions in this signaling pathway.

Autophagy is one of the means by which a cell maintains its normal functioning by degrading damaged organelles, misfolded/unwanted proteins as well as invading pathogens by trapping them in a double membrane structure called autophagosome and delivering them to the vacuole/lysosome for degradation by acid hydrolases [111]. This process is highly conserved in eukaryotes and is important for stress tolerance and nutrient-induced signaling [112]. Recently, some literature emerged linking retromer function to autophagic processes, though its role may be an indirect one or may be dispensable for autophagy in some cells [113]. WASH complex regulates the trafficking of an essential autophagic protein, Atg9, to the autophagosome; Vps35 (D620N) mutation blocks this transport, which results in inhibition of autophagy [114]. Depletion of retromers in fat body cells of *Drosophila* also perturbs autophagy, and results in accumulation of undigested cytoplasmic and endosomal contents in autophagosomes [115]. Our research group previously reported the involvement of retromer complex in the trafficking of *Magnaporthe oryzae* Atg8 (MoAtg8), where loss of retromer function causes vacuolar degradation of this protein and impairs autophagosome biogenesis, which blocks programmed cell death (PCD) in appressorial formation from *M. oryzae* conidia [112]. Of all the identified autophagy genes, only Atg8 and Atg9 gene products have so far been reported to be retromer-dependent for their function. Further investigations are therefore required to understand retromer function in relation to autophagy.

## 7. Retromer and Invading Pathogens

In pathogens, different strategies have evolved that ensure successful host colonization. Some use, to their advantage, their host cell’s retromer function to achieve invasion as seen in HPV and HIV-1 [12,116], whereas others either by-pass or inhibit retromer. Influenza A virus produces a matrix protein 2 (M2) essential for its replication [117]. M2 uses retromer to escape degradation by being transported from the early endosome to the TGN, where it is translocated to the ER for release into the cytosol [118]. Similarly, hepatitis C virus (HCV) diverts retromer and its cargo, CI-MPR, to its replication site and uses them for its replication; silencing of both retromer and CI-MPR inhibits and reduces HCV replication, respectively [119]. Interaction of the retromer with autophagosome also promotes HCV replication [120], but further work is needed to establish how HCV uses the advantage of this interaction.

Moreover, *Shigella dysenteriae* and some serotypes of *Escherichia coli* secrete shiga toxin (Stx) and shiga-like toxin, respectively, into their hosts where the toxins “disguise” as retromer cargoes at the late endosome, to be transported to the TGN and then to the ER where they inhibit the host cell’s protein translation [121]. The use of retromer function by the protozoan pathogen, *Toxoplasma gondii*, to avoid the degradation pathway has been comprehensively reviewed [122].

While all the aforesaid pathogens utilize retromer function to their advantage, some other pathogens may find retromer activity a hurdle to invading their hosts. *Chlamydia trachomatis*, an important sexually transmitted bacterium, replicates in a vacuole called the inclusion (Inc) within an infected cell [123]. A protein on the Inc surface, IncE, binds a conserved region on SNX5 that prevents CI-MPR sorting [42]. The consequence of this is the malfunctioning of the lysosome due to blockage in the supply of hydrolytic enzymes. Growth of *C. trachomatis* infectious progeny increased significantly in SNX5/SNX6 depleted cells [124,125]. However, the link between lysosomal function and *Chlamydia* replication is still unclear. Perhaps, accumulation of cholesterol at the late endosome due to disruption of CI-MPR recycling [126] or perturbation of retromer function [104] provides sufficient nutrients for *C. trachomatis* development. Another pathogen, *Legionella pneumophila* (a causative agent of some pneumonia), also replicates best in the absence of retromer function [127]. Its effector protein, RidL, binds Vps29 and PI(3)P to inhibit retromer function for successful replication of the pathogen [128]. Collectively, invading pathogens manipulate retromer either by using the advantage of its function or by inhibiting its activities to achieve colonization.

## 8. Retromer Dysfunction

Considering the central role played by the retromer complex in protein trafficking, one should expect serious pathogeneses resulting from its malfunction. Loss of any component of the CSC has been shown to attenuate retromer function [57,106,109,129]. Retromer dysfunction may result, at least, from mutations of these components, particularly Vps35. The most studied retromer dysfunction-related pathogeneses are neurodegenerative disorders including Alzheimer’s and Parkinson’s diseases. Alzheimer’s disease (AD) is characterized by loss of memory, which progresses with age. A lot of factors, both genetic and otherwise, lead to development of this disorder, which makes it difficult to pinpoint a single cause of the disease. However, retromer dysfunction is an important contributing factor as decreased levels of Vps35 and Vps26 were observed in patients with late onset of the disease [130]. Consistent with this, Chu and Praticò discovered that the expression levels of Vps35 and Vps26 (but not Vps29) decreased with age in the cortex (a vulnerable region to neurodegeneration) of Tg2576 transgenic mice model of AD, but remained unaltered in the cerebellum [131]. More so, they found that the levels of the CSC-binding cargoes, CI-MPR and SorLA, were also significantly reduced in the cortex with age. Amyloid precursor protein (APP) is a transmembrane protein whose cleavage by β secretase leads to amyloid-beta (Aβ) production in neurons [132]. APP is a retromer cargo [8,133] whose mistrafficking due to retromer failure causes accumulation of Aβ in the endosome leading to neuronal dysfunction, hence AD progression [134,135].

Parkinson’s disease (PD), on the other hand, is characterized by accumulation of Lewy Bodies due to elevated levels of undegraded α-synuclein in the endosome, which leads to neuron degeneration. Cathepsin D is transported from the Golgi to the early endosome by CI-MPR where it is finally delivered to the lysosome to degrade α-synuclein [136]. Retromer dysfunction prevents CI-MPR recycling, which therefore causes α-synuclein accumulation. α-synuclein-mediated toxicity was investigated in yeast and was shown to develop in the absence of Vps35 [137]. Interestingly, α-synuclein interacts with the retromer complex, though it still has an unknown function [138]. Moreover, aggregation of α-synuclein was also observed in Vps35 (R524W)-containing retromer (but not Vps35 (P316S)-containing retromer) and this impairs retromer association with its regulating machineries, delaying retrograde trafficking but not SNX27-retromer-dependent recycling [70]. The observed defects in PD were shown to develop due to a decrease in the affinity of Vps35 for FAM21 in Vps35 (D620N)-expressing cells [43]. One of the critical physiologic problems in PD is mitochondrial fragmentation resulting from enhanced Vps35–DLP (dynamin-like protein) interaction [139]. In summary, retromer dysfunction is one of the principal causes of AD, PD, and other related neurodegenerative disorders.

## 9. Other Physiological Roles of the Retromer

The retromer complex regulates many physiological processes involving different biochemical pathways. These include the regulation of morphogenesis by Chs3, which maintains yeast cell integrity via cell wall remodeling in different environmental conditions [140]. Internalized Chs3 has been shown to escape vacuolar degradation via retromer-dependent sorting and trafficking to the TGN where it is recycled to the plasma membrane [105]. In addition to Chs3, three novel retromer cargoes, Ato3, Ymd8, and Ymr253c, have recently been identified in yeast [59], though their trafficking pathways are still unknown. Furthermore, our group previously investigated the role of retromers in the model fungus, *Fusarium graminearum*, using combined cell biological and genomics approaches [141]. We showed that the normal growth, conidiation, ascospore formation, and pathogenicity of *F. graminearum* require retromer activity. The cargoes that may relate the retromer to these development-related processes are, however, yet to be identified.

Moreover, phospholipase D (PLD) was shown to be involved in cell clearance of rhodopsin1-containing vesicles (RLVs), a critical process in preventing retinal degeneration during illumination in *Drosophila* photoreceptors [13]. The report indicated that PLD activity is retromer-dependent as RNAi suppression of Vps35 function resulted in an elevated number of RLVs in Rab7-positive compartments, but the mechanistic link between PLD activity and retromer awaits further clarification.

Triggering receptor expressed on myeloid cells 2 (Trem2) is a transmembrane glycoprotein important for modulating innate immune system [142]. After its clathrin-dependent endocytosis, this protein requires retromer for its endosome-to-TGN trafficking and cells expressing Vps35 (R47H) mutation accumulated Trem2 at the endosome [15]. The role of retromers in polarized protein trafficking has recently been comprehensively reviewed [143]. Lastly, depletion of retromers in hippocampal neurons blocks long-term potentiation (LTP) due to mistrafficking of AMPA (α-amino-3-hydroxy-5-methyl-4-isoxazolepropionic acid) and this has been linked to loss of memory in both AD and PD patients [144]. Retromer is involved in many physiological processes some of which are not clearly understood. Further clarifications of its physiological roles will hopefully improve our understanding of many complex cellular processes.

## 10. Conclusions and Future Perspectives

The capacity of a cell to respond appropriately to both environmental and metabolic stimulants is a major feature that defines its survival competence and maintenance of its functions. Intracellular trafficking is thus required for normal homeostasis in eukaryotic cells. Substantial discoveries have been made in recent years on the structural and functional roles of this protein complex, which may differ in different species. In this review, we present a generalized overview of progress, including (but not limited to) the following findings:

- Retromer-mediated cargo sorting at the vacuole and its transport back to the endosomes;
- A consensus sorting signal, (F/Y)E(F/L), for SNX3-retromer;
- Retromer cargoes taking different routes leave the endosomes in a shared vesicle before further fission of the vesicle;
- Dependence of SNX27-retromer cargo routing on FAM21 but not on other WASH components (strumpellin and WASH1);
- Disruption of retromer-dependent trafficking (but not SNX27-retromer-dependent recycling) due to Vps35(R524W) mutation;
- Regulation of the release of SNX27-retromer cargoes by a novel protein, ANKRD50;
- Involvement of all the retromer forms in PTHR recycling;
- Timely regulation of retromer coating and uncoating of vesicles by TBC1D5 for cargo sorting and trafficking;
- Essential role of Mih1 signaling in phosphorylation of the 6 loop of Vps26, which in turn regulates retromer-dependent recycling of Chs3;
- Termination of PTH-PTHR generated cAMP signaling as well as that of JAK/STAT signaling by retromer complex;
- Retromer regulation of autophagy in *Drosophila* and *M. oryzae*;
- Manipulation of retromer by HPV, HIV-1, HCV and influenza A virus to achieve host cell invasion;
- Age-dependent expression of *VPS35* and *VPS26* genes in the cortex of an Alzheimer’s disease rat model;
- Possible mitochondrial fragmentation in Parkinson’s disease patients due to retromer dysfunction;
- Ato3, Ymd8, and Ymr253c as novel retromer cargoes in yeast.

Dissecting the mechanism of action of retromer complex in its different pathways, uncovering the determining factors of its trafficking route, as well as the cargo specificity of the different retromer forms will hopefully unveil new insights into cargo sorting and orchestration and regulation of physiological processes in addition to developing new strategies for diagnosis and treatment of some human diseases.
